# Effects of cognitive behavioral therapy on sleep quality and insomnia severity index in women with menopausal insomnia: a systematic review and meta-analysis

**DOI:** 10.4069/whn.2025.09.07

**Published:** 2025-12-31

**Authors:** Hee-Jin Moon, Se-Na Yu, Myung-Haeng Hur

**Affiliations:** 1 Department of Nursing, Eulji University Hospital, Uijeongbu, Korea; 2 Department of Nursing. Nowon Eulji University Hospital, Seoul, Korea; 3 College of Nursing, Eulji University, Uijeongbu, Korea

**Keywords:** Cognitive behavioral therapy, Insomnia, Menopause, Meta-analysis, Sleep quality

## Abstract

**Purpose:**

This study aimed to evaluate the effects of cognitive behavioral therapy for insomnia (CBT-I) on sleep quality and insomnia severity in menopausal women through a systematic review and meta-analysis.

**Methods:**

A comprehensive literature search was conducted up to October 2024 using PubMed, EMBASE, Cochrane, and CINAHL, with additional searches of Chinese and Korean databases to include East Asian studies. Randomized controlled trials (RCTs) assessing the effects of CBT-I on sleep outcomes in menopausal women were included. Eleven RCTs (n=973) met the inclusion criteria. The interventions comprised face-to-face, telephone, and internet-based CBT-I programs, with session counts ranging from 4 to 12 and follow-up durations extending from post-intervention to 52 weeks. Data were analyzed using Review Manager 5.4, and effect sizes were expressed as standardized mean differences (SMDs) and mean differences (MDs) with 95% confidence intervals (CIs).

**Results:**

CBT-I significantly improved sleep quality (n=795, SMD=−1.01; 95% CI, −1.27 to −0.75) and reduced insomnia severity (n=504, MD=−4.49; 95% CI, −6.12 to −2.87). Subgroup analyses indicated that CBT-I was effective regardless of delivery mode (face-to-face or remote), follow-up duration, or baseline insomnia severity.

**Conclusion:**

CBT-I is an effective non-pharmacological intervention for improving sleep quality and reducing insomnia severity in menopausal women. These findings support the integration of CBT-I into clinical practice, particularly as a nurse-led intervention that can be delivered in both face-to-face and remote formats. To enable broader implementation, standardized CBT-I training programs and clinical protocols should be developed. Future studies should investigate long-term effectiveness and cultural applicability in diverse populations, including Korean women.

## Introduction

Menopause is a complex, natural physiological transition in a woman’s life, characterized by substantial hormonal changes, particularly declines in estrogen and progesterone levels [1]. These hormonal fluctuations are strongly associated with vasomotor symptoms such as hot flashes and night sweats, which frequently disrupt sleep continuity [2,3]. Nocturnal hot flashes, in particular, can cause repeated awakenings and difficulty returning to sleep, leading to fragmented sleep and reduced sleep efficiency.

In addition to these biological disturbances, psychological challenges—such as mood instability, anxiety, depressive symptoms, and role-related stress—are also common during menopause [4,5]. These interrelated biological and psychological factors contribute to the high prevalence and persistence of insomnia in menopausal women. Although insomnia is frequent among middle-aged adults in general, the combination of hormonal changes, vasomotor instability, and psychosocial stressors in menopausal women underscores the need for targeted treatment strategies. Understanding these mechanisms is essential for developing effective clinical approaches to manage menopausal insomnia.

Numerous studies worldwide have examined both pharmacological and non-pharmacological interventions for insomnia [6,7]. Pharmacological approaches often provide immediate relief; however, they can result in rebound insomnia after discontinuation and may increase dependence on medication. Additionally, the potential for adverse side effects makes such interventions less desirable as a first-line treatment [8]. Consequently, many patients prefer non-pharmacological strategies, and various studies have shown that their effectiveness is comparable to that of pharmacological treatments [9,10].

Non-pharmacological interventions for insomnia include cognitive behavioral therapy (CBT), relaxation and meditation techniques, regular exercise, circadian rhythm regulation, sleep diaries, aromatherapy, acupuncture, and music therapy [10]. Among these, CBT for insomnia (CBT-I) is widely recognized as an evidence-based first-line treatment for insomnia.

CBT is a form of psychotherapy based on the principle that thoughts, emotions, and behaviors are interrelated and mutually influential. It is a structured, short-term, and empirically supported approach proven effective for a range of psychological conditions, including depression, anxiety disorders, obsessive-compulsive disorder, panic disorder, and post-traumatic stress disorder [11]. CBT involves identifying core problems, analyzing dysfunctional thought and behavior patterns, learning coping and relaxation techniques, gradually confronting anxiety-provoking situations, and evaluating progress toward treatment goals [12]. This structured and goal-oriented process is especially effective for individuals who are aware of their difficulties and motivated to change.

Building on the cognitive restructuring framework of CBT, CBT-I focuses on identifying and modifying maladaptive thoughts and behaviors that perpetuate insomnia. It has demonstrated both short-term efficacy and long-term sustainability, making it a highly effective treatment without the side effects associated with pharmacotherapy. The main components of CBT-I include sleep restriction therapy, stimulus control therapy, sleep hygiene education, and cognitive therapy [13].

Sleep restriction therapy adjusts bedtime and wake time to match the average actual sleep duration, thereby minimizing the discrepancy between time spent in bed and total sleep time. Stimulus control therapy promotes healthy sleep habits by encouraging individuals to use the bed only for sleep or sexual activity, to leave the bed if unable to fall asleep within 15–20 minutes, and to maintain consistent wake times without daytime naps. Sleep hygiene education provides behavioral guidance and practical information to support better sleep routines, while cognitive therapy targets dysfunctional beliefs and attitudes about sleep, helping individuals restructure their cognitive approach toward rest [13,14].

CBT-I has been shown to significantly improve insomnia symptoms. Although studies in other countries have actively examined remote delivery methods such as telephone- or internet-based CBT-I, particularly during the COVID-19 pandemic, research in Korea remains limited. In particular, few studies have focused specifically on CBT-I for menopausal insomnia [2,15].

According to the Korea National Health and Nutrition Examination Survey, sleep disturbances are a major factor affecting subjective health and health-related quality of life among middle-aged women aged 40 to 59 years. As the number of menopausal women experiencing insomnia continues to rise in Korea, there is an increasing need for research on sustainable, non-pharmacological treatment strategies [16].

Therefore, evaluating the specific effectiveness of CBT-I is crucial for developing evidence-based interventions to manage menopausal insomnia. Previous studies have reported significant improvements when CBT-I was applied alone or in combination with pharmacological or other non-pharmacological approaches [2,4]. Based on this evidence, the present study aims to assess the effectiveness of CBT-I in improving sleep quality and reducing insomnia severity among menopausal women.

## Methods

**Ethics Statement:** The requirement to obtain informed consent was exempted by the Institutional Review Board of Eulji University (No. EUIRB2024-097) because there was no sensitive information and the survey was anonymously treated.

### Study design

This study is a systematic review and meta-analysis conducted to objectively evaluate the effects of CBT-I on menopausal insomnia by analyzing randomized controlled trials (RCTs). The study was registered with PROSPERO (CRD42024604013) and was reported in accordance with the PRISMA 2020 guidelines [17].

### Inclusion and exclusion criteria

The patients, intervention, comparison, outcome, and study design (PICOS) framework was used to structure the clinical question for this systematic review. The patients (P) were: (1) women aged 40–60 years in the early to late stages of menopause, and (2) those who, by self-report, experienced early, middle, or late insomnia at least three times per week for more than 3 months despite having an adequate sleep environment, resulting in functional impairment; or those with an Insomnia Severity Index (ISI) score ≥7 or a Pittsburgh Sleep Quality Index (PSQI) score ≥5. The interventions (I) involved CBT-I, including components such as stimulus control therapy, sleep restriction therapy, cognitive therapy, and sleep hygiene education. The comparison (C) group consisted of participants who received no intervention or routine care, such as general menopausal care, menopause education, or sleep hygiene education. The outcomes (O) included sleep quality as the primary outcome and insomnia severity as the secondary outcome. The study design (S) was limited to RCTs.

### Search strategy

A comprehensive literature search and analysis were conducted for studies published up to October 2024. The international databases searched included PubMed, EMBASE, the Cochrane Central Register of Controlled Trials (CENTRAL), and CINAHL. To include East Asian studies, regional databases from China and Korea were also searched. The Chinese databases included the China National Knowledge Infrastructure (CNKI), VIP (Chinese Science and Technology Journals Database), and Wanfang (Chinese academic journals and theses). The Korean databases included RISS (academic research portal), NDSL (science and technology literature), DBpia (journal articles and theses), KISS (multidisciplinary academic literature), and ScienceON (science and technology database). Both peer-reviewed journal articles and academic theses were retrieved. Studies published in English, Korean, and Chinese were included to ensure comprehensive coverage, as research on menopausal insomnia and CBT-I has been actively conducted in these languages. To enhance search sensitivity, gray literature—including theses, reports, and conference presentations—was manually searched using Google Scholar in addition to the electronic databases. Search terms were developed using a combination of MeSH terms and free-text keywords, applying Boolean operators (AND/OR) and truncation where appropriate. For Participants (P), the keywords included “Insomnia,” “Sleep,” “Disorder,” “Problem,” “Menopause,”* and “Middle age.” For Interventions (I), the terms “Cognitive behavioral therapy [MeSH],” “Cognitive behavior therapy,” and “CBT” were used. For Outcomes (O), the keywords “Sleep quality,” “PSQI,” “Pittsburgh Sleep Quality Index,” “VSH,” “Verran and Snyder-Halpern,” “ISI,” and “Insomnia Severity Index” were applied. The final search string was constructed using the following structure: Participants: ((Insomnia) OR ((Sleep) AND ((Disorder) OR (Problem)))) AND ((Menopause*) OR (Middle age)), Intervention: (Cognitive behavioral therapy) OR (Cognitive behavior therapy) OR (CBT), Outcome: (Sleep quality) OR (PSQI) OR (Pittsburgh Sleep Quality Index) OR (VSH) OR (Verran and Snyder‐Halpern) OR (ISI) OR (Insomnia Severity Index). Advanced search options were used where available to optimize search sensitivity and specificity according to the features of each database (Supplementary Table 1).

### Selection process of studies

The inclusion criteria for this review were: (1) intervention studies examining the effects of CBT-I on menopausal insomnia; (2) RCTs; (3) studies in which the control group either received no intervention or only general education, allowing the specific effect of CBT-I to be isolated; and (4) studies where the intervention provided to the control group was also applied to the experimental group, ensuring comparability between conditions. The exclusion criteria were: (1) preclinical or animal studies and studies involving children; (2) studies not published in Korean, English, or Chinese; (3) observational studies or review articles; (4) studies incorporating additional pharmacological interventions beyond participants’ usual medications; and (5) studies in which the interventions for the experimental and control groups were not equivalent, making it impossible to isolate the specific effects of CBT-I. No disagreements occurred between the researchers during study selection.

**Figure 1.**
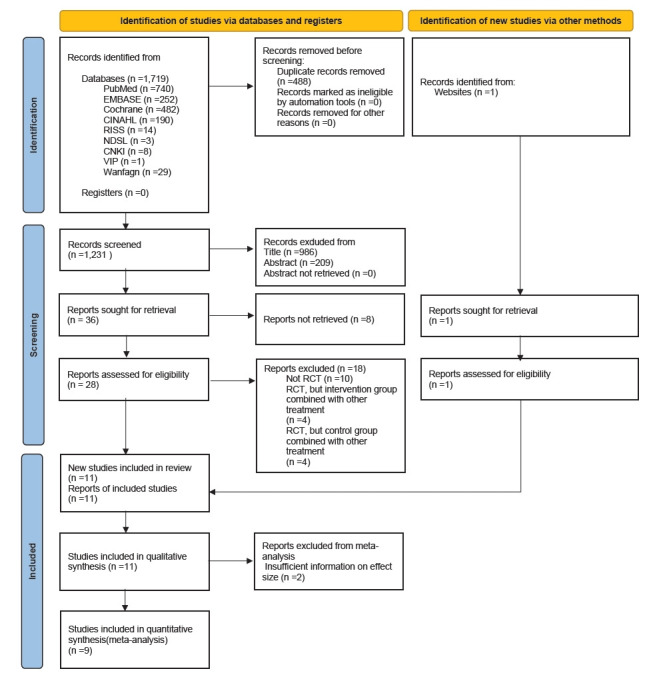
PRISMA 2020 flow chart of study selection process. RCT: Randomized controlled trial.

Studies were selected based on the core research question and the predefined inclusion and exclusion criteria. The PRISMA 2020 flow diagram (Figure 1) presents the detailed study selection process. A total of 1,720 records were initially retrieved through the search strategy. After removing 488 duplicates, 1,232 articles were screened by two researchers (authors HJM and MHH) according to the inclusion and exclusion criteria. As a result, 1,195 articles that did not meet the criteria were excluded, and 36 studies were initially selected for full-text review. Of these, eight were excluded because they were unpublished, available only as abstracts, or lacked sufficient methodological detail. The remaining 29 articles were re-evaluated by two researchers using the same criteria. Among these, 18 were excluded because they were not RCTs, did not apply a single intervention to the experimental group, or did not apply a uniform control condition to the comparison group. Ultimately, 11 studies [3,8-25] were included in the qualitative synthesis. Of these, nine studies were incorporated into the meta-analysis, as they employed consistent intervention methods and outcome variables, clearly reported sample sizes for both groups, and provided post-intervention outcome data suitable for quantitative analysis.

### Quality assessment

The methodological quality of the included RCTs was evaluated using the Risk of Bias 2.0 (RoB 2.0) tool developed by the Cochrane Collaboration [26]. This updated instrument assesses the potential for bias across five core domains that may influence study outcomes: (1) bias arising from the randomization process, (2) bias due to deviations from intended interventions, (3) bias due to missing outcome data, (4) bias in the measurement of outcomes, and (5) bias in the selection of reported results.

Each domain includes a structured set of signaling questions, with responses categorized as “yes,” “probably yes,” “probably no,” “no,” or “no information.” An embedded algorithm interprets these responses to classify the risk of bias for each domain as low risk, some concerns, or high risk. The overall risk of bias for each study was determined by aggregating judgments across the five domains: studies rated as low risk in all domains were classified as having a “low overall risk”; studies with at least one domain rated as “some concerns” were categorized accordingly; and those with one or more domains rated as “high risk” were classified as having a “high overall risk of bias” [27].

Reviewers (authors HJM, SNY, and MHH) independently assessed each study using the RoB 2.0 tool. Any discrepancies between reviewers were resolved through discussion and consensus. This process ensured methodological rigor and reliability in quality assessment. The results of the evaluations were visualized using the robvis tool (http://mchuinlu.shinyapps.io/robvis/).

### Data extraction and analysis

The characteristics of the studies included in this systematic review were analyzed, and data were extracted based on the following variables: first author, publication year, country, sample size, participant age, type of intervention, type of control intervention, primary outcome variables, and the authors’ conclusions (Table 1).

### Model selection

Given the observed heterogeneity among studies in intervention methods, duration, and insomnia severity, a random-effects model was employed to account for both within- and between-study variance.

### Estimation of effect size

Data were analyzed using Review Manager (RevMan) version 5.4 (Cochrane Collaboration). As the outcomes reported in the included studies were continuous variables, effect sizes were calculated using the number of participants (n), mean values, and standard deviations for both intervention and control groups. For insomnia severity, all studies utilized a consistent measurement instrument—the ISI—allowing for the calculation of mean differences (MDs) between groups. In contrast, sleep quality was assessed using various validated instruments, including the PSQI, Groningen Sleep Quality Scale, and others; therefore, standardized mean differences (SMDs) were calculated to synthesize these outcomes.

## Results

### Study characteristics

Among the 11 included studies, approximately half were conducted in the United States [3,14,15,18-25], while the others were conducted in Egypt [15,18], China [3,21], Iran [19], and South Korea [20]. About half of the studies were published before 2020 [22-25], with the remainder published thereafter. As presented in Table 2, face-to-face CBT interventions were predominant, being applied in eight studies [3,14,18-23], whereas two studies employed remote delivery [15,25] and one study utilized both delivery modes [24]. The number of CBT sessions ranged from 4 to 12, with most delivered once per week. Session duration varied from 20 to 120 minutes, with 50–60 minutes being the most common; four studies [3,14,21,23] did not report session length. Follow-up periods ranged from immediate post-intervention to 52 weeks.

### Quality assessment of selected studies

**Figure 2.**
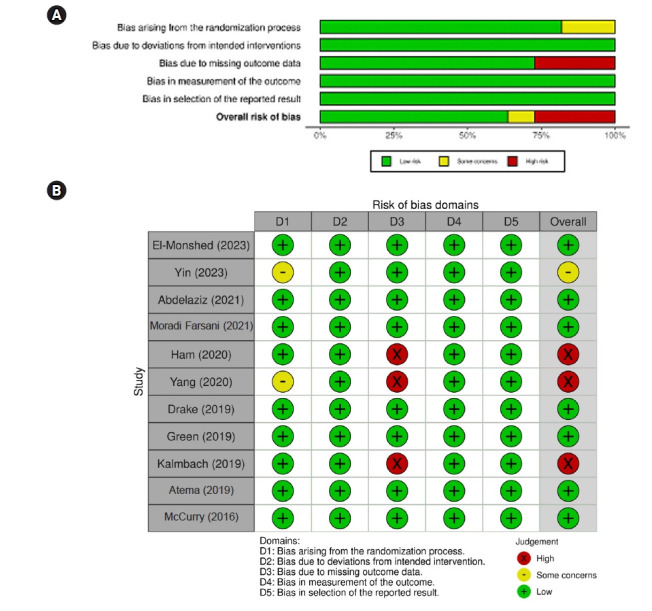
Risk of bias in included studies. (A) Risk of bias graph. (B) Risk of bias of selected studies.

As shown in Figure 2, the risk of bias arising from the randomization process was rated as low in nine studies (81.8%) [14,15,18-20,22-25] and as having some concerns in two studies (18.2%) [3,21]. For bias due to missing outcome data, eight studies (72.7%) [3,14,15,18,19,22,24,25] were rated as low risk, while three studies (27.3%) [20,21,24] were assessed as high risk. Bias related to deviations from intended interventions, outcome measurement, and selection of reported results was rated as low risk across all included studies.

Overall, the methodological quality of the included studies was considered acceptable, with most domains demonstrating a low risk of bias. However, the presence of high risk in the domain of missing outcome data in three studies suggests that some caution is warranted when interpreting the pooled findings. Despite this, the consistency of results across studies with low risk supports the robustness of the meta-analytic conclusions.

### Effectiveness of intervention studies

#### Effects of CBT-I on sleep quality in menopause

##### 1) Effect size of CBT-I on sleep quality

**Figure 3.**
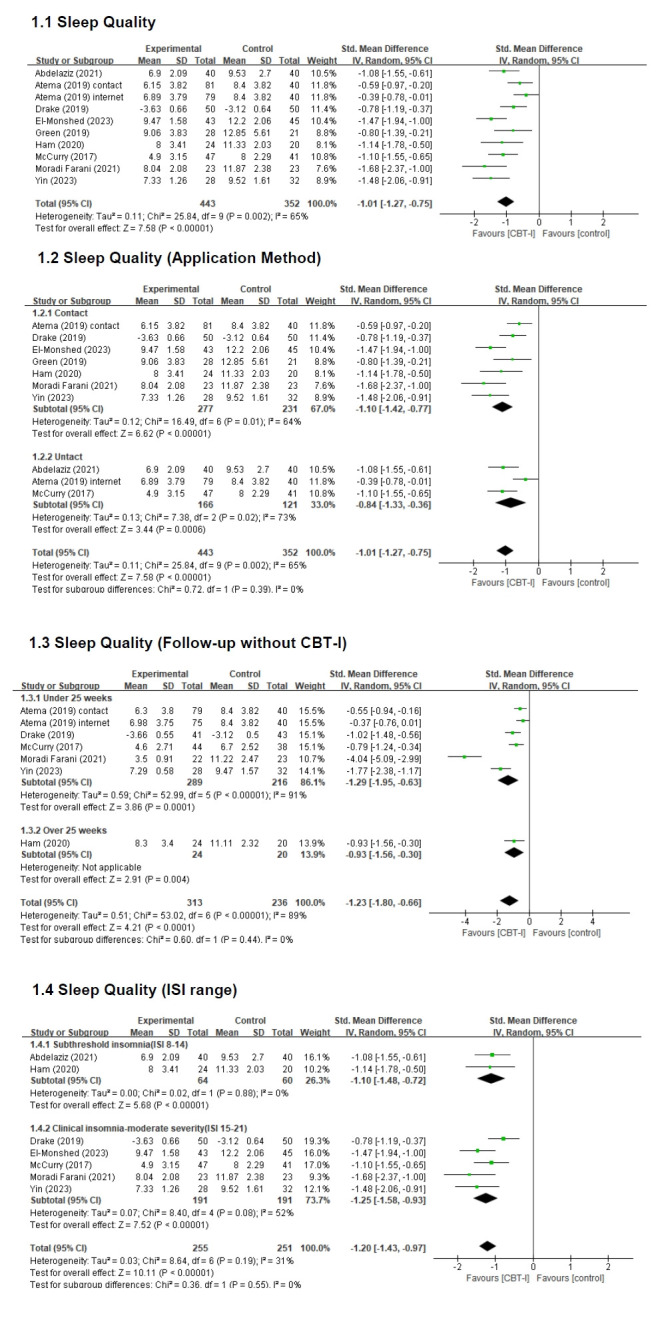
Forest plot of sleep quality. CBT-I: Cognitive behavioral therapy for insomnia; CI: confidence interval; df: degree of freedom; ISI: Insomnia Severity Index; SD: standard deviation.

The SMD between the intervention and control groups for the effect of CBT-I on sleep quality was −1.01 (n=795; 95% CI −1.27 to −0.75), indicating a significant improvement in sleep quality. This effect was statistically significant (Z=7.58, *p*<.001). Assessment of heterogeneity revealed moderate heterogeneity among studies (Higgins I^2^=65%); therefore, a random-effects model was applied to analyze the effect size of CBT-I on sleep quality (Figure 3).

##### 2) Effect size according to the application method of CBT-I

For face-to-face CBT-I, the intervention group demonstrated a significant improvement in sleep quality compared with the control group (n=508, SMD=−1.10; 95% CI, −1.42 to −0.77), and this difference was statistically significant (Z=6.62, *p*<.001). Similarly, for remotely delivered CBT-I, the intervention group also exhibited a significant improvement over the control group (n=287, SMD=−0.84; 95% CI, −1.33 to −0.36), which was statistically significant (Z=3.44, *p*<.001). However, the difference in effect size between face-to-face and remote CBT-I delivery was not statistically significant (*p*=.39) (Figure 3).

##### 3) Effect size according to follow-up without CBT-I

When the follow-up period without CBT-I was less than 25 weeks, the intervention group showed a greater improvement in sleep quality than the control group (n=505, SMD=−1.29; 95% CI, −1.95 to −0.63), with a statistically significant difference (Z=3.86, *p*<.001). When the follow-up period exceeded 25 weeks, the intervention group continued to show significant improvement compared to the control group (n=44, SMD=−0.93; 95% CI, −1.56 to −0.30), and this difference was statistically significant (Z=2.91, *p*<.01). However, the difference in effect size between the groups reassessed within 25 weeks and those reassessed after 25 weeks was not statistically significant (*p*=.44) (Figure 3).

##### 4) Effect size of CBT-I according to ISI range

For participants with subthreshold insomnia (ISI score, 8–14), CBT-I significantly improved sleep quality compared to the control group (n=124, SMD=−1.10; 95% CI, −1.48 to −0.72), and this effect was statistically significant (Z=5.68, *p*<.001). Among participants with clinical insomnia (ISI score, 15–21), CBT-I also significantly improved sleep quality (n=382, SMD=−1.25; 95% CI, −1.58 to −0.93), with a statistically significant difference (Z=7.52, *p*<.001). However, the difference in effect size between the subthreshold and clinical insomnia groups was not statistically significant (*p*=.55) (Figure 3).

#### Effects of CBT-I on ISI in menopause

##### 1) Effect size of CBT-I on ISI

**Figure 4.**
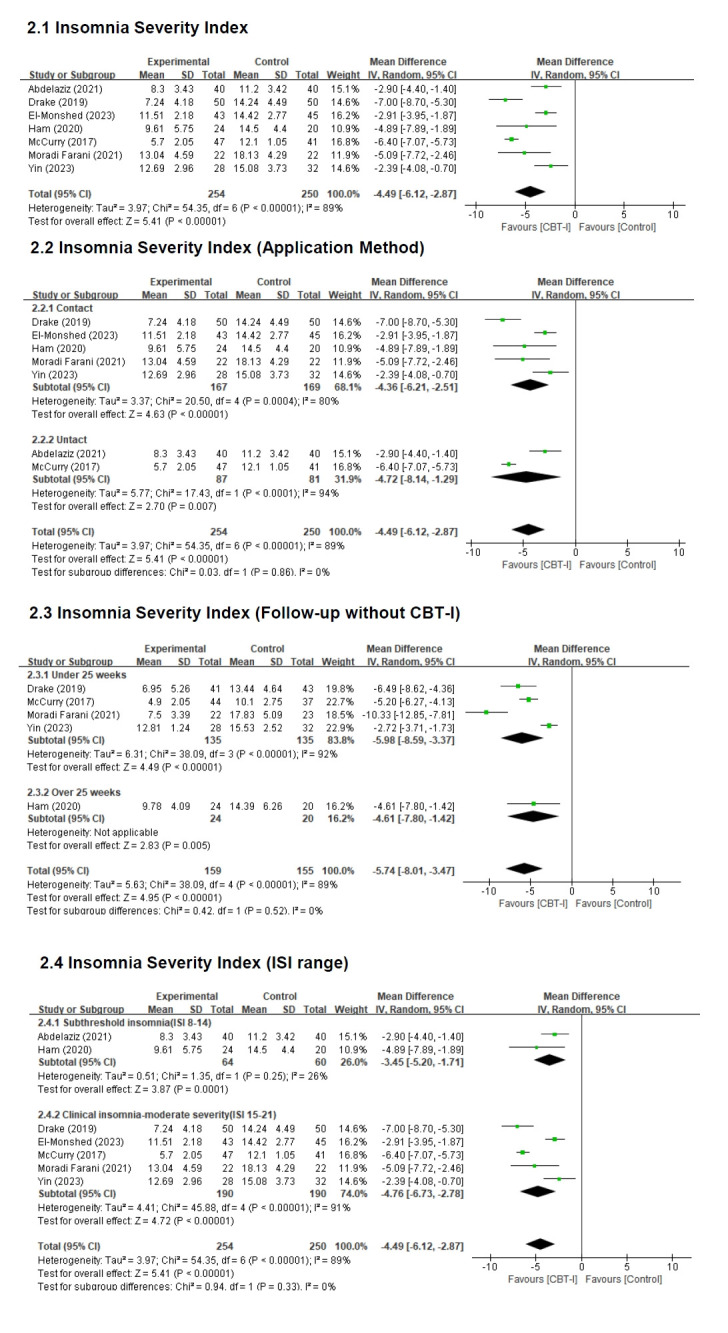
Forest plot of Insomnia Severity Index. CBT-I: Cognitive behavioral therapy for insomnia; CI: confidence interval; df: degree of freedom; ISI: Insomnia Severity Index; SD: standard deviation.

The effect size of CBT-I on insomnia severity demonstrated a significant reduction in the intervention group compared with the control group, with an MD of −4.49 points (n=504; 95% CI, −6.12 to −2.87). This difference was statistically significant (Z=5.41, *p*<.001). Heterogeneity across studies was high (Higgins I²=89%); therefore, a random-effects model was applied to analyze the effect size of CBT-I on sleep quality (Figure 4).

##### 2) Effect size according to the application method of CBT-I

For insomnia severity, face-to-face CBT-I resulted in a mean reduction of 4.36 points in the intervention group compared with the control group (n=326; MD=−4.36; 95% CI, −6.21 to −2.51), which was statistically significant (Z=4.63, *p*<.001). Non-face-to-face CBT-I produced a mean reduction of 4.72 points in the intervention group compared with the control group (n=168; MD=−4.72; 95% CI, −8.14 to −1.29), and this difference was also statistically significant (Z=2.70, *p*<.01). However, the difference in effect size between face-to-face and non-face-to-face CBT-I was not statistically significant (*p*=.86) (Figure 4).

##### 3) Effect size according to follow-up without CBT-I

When the follow-up period after CBT-I was less than 25 weeks, the intervention group exhibited a significant 5.98-point reduction in insomnia severity compared with the control group (n=270; MD=−5.98; 95% CI, −8.59 to −3.37), and this difference was statistically significant (Z=4.49, *p*<.001). For follow-up periods between 25 and 52 weeks, the effect size indicated a 4.61-point reduction in insomnia severity in the intervention group compared with the control group (n=44; MD=−4.61; 95% CI, −7.80 to −1.42), which was also statistically significant (Z=2.83, *p*<.01). However, the difference in effect size between studies with follow-up periods of less than 25 weeks and those exceeding 25 weeks was not statistically significant (*p*=.52) (Figure 4).

##### 4) Effect size of CBT-I according to ISI range

Among participants with subthreshold insomnia (ISI score, 8–14), CBT-I significantly reduced insomnia severity compared with the control group, with a mean difference of −3.45 points (n=124; 95% CI, −5.20 to −1.71), and this difference was statistically significant (Z=3.87, *p*<.001). For participants with clinical insomnia (ISI, 15–21), CBT-I also significantly reduced insomnia severity (n=380; MD=−4.76; 95% CI, −6.73 to −2.78), with a statistically significant difference (Z=4.72, *p*<.001). However, the difference in effect size between the subthreshold and clinical insomnia groups was not statistically significant (*p*=.33) (Figure 4).

## Discussion

Beyond statistical significance, the effectiveness of CBT-I may stem from its ability to address both biological and psychological factors contributing to menopausal insomnia [2,13,25]. CBT-I promotes long-term behavioral change by helping individuals restructure maladaptive thoughts and beliefs, which can lead to sustained improvements in sleep even after treatment completion. This may explain why both in-person and remote CBT-I formats demonstrated similar effectiveness, potentially reflecting comparable levels of therapeutic engagement, participant motivation, and fidelity in program implementation.

Of the nine studies included in this review, one employed both in-person and remote CBT-I interventions with a control group. For meta-analytic purposes, this study was divided, resulting in 10 total comparisons. The meta-analysis showed that CBT-I significantly improved sleep quality compared with control groups. Subgroup analyses were conducted according to delivery method (in-person vs. remote), follow-up period, and baseline ISI scores. All subgroups demonstrated statistically significant improvements, consistent with prior findings [4,28].

Notably, no significant difference was observed between in-person and remote interventions, aligning with earlier research such as studies on internet-based CBT-I for breast cancer survivors [24] and sleep clinic patients (digital CBT-I: ISI 19.4 → 12.3; in-person CBT-I: ISI 18.4 → 8.9) [29]. Likewise, internet-based CBT-I for menopausal [15] and adult populations [30] yielded positive outcomes over 6 to 9 weeks. Given the relatively long duration of CBT-I interventions, remote delivery provides a practical and cost-effective alternative. However, although no significant difference was detected between delivery formats in the meta-analysis, the remote CBT-I studies varied substantially in design (e.g., internet-based, mobile app-based, or telehealth platforms), which may have introduced heterogeneity. This variability should be considered when interpreting the results.

Furthermore, remotely delivered CBT-I can enhance accessibility for women who encounter barriers such as limited mobility, time constraints, or geographic isolation [15,24,25]. Partnerships with community-based organizations, public health centers, and primary care clinics could further extend the reach and sustainability of these programs. Subgroup analysis by follow-up duration (≤25 weeks vs. >25 weeks) demonstrated that CBT-I maintained effectiveness regardless of time elapsed, contrasting with the waning effects often observed in pharmacologic or exercise-based therapies. This durability likely reflects the core components of CBT-I—sleep restriction, stimulus control, cognitive therapy, and sleep hygiene education—which reinforce lasting behavioral change.

Additionally, CBT-I was effective for both subthreshold (ISI score, 8–14) and clinical insomnia (ISI score, 15–21). Previous studies have reported significant ISI reductions following face-to-face CBT-I among college students [31] and after internet-based CBT-I among adults (predominantly female, 77.5%; with menopausal status not specified) [32]. The current findings corroborate CBT-I’s efficacy across severity levels, though further large-scale, high-quality studies are warranted to validate these results. Moreover, approximately half of the included studies were conducted in the United States, while the remainder originated from Egypt, South Korea, and China. Cultural differences in perceptions of sleep, mental health, and menopause may influence intervention uptake and response, potentially limiting generalizability. Future research should therefore explore the cultural adaptability and contextual relevance of CBT-I across different cultural contexts other than those represented in the included studies.

In conclusion, CBT-I is effective in improving sleep quality and reducing insomnia severity among menopausal women, regardless of delivery format, intervention duration, or baseline severity. Comparable outcomes between face-to-face and remote CBT-I highlight remote delivery as a viable, cost-effective approach. Empowering women’s health nurses through standardized CBT-I training and institutional support can facilitate broader implementation, expanding access to holistic, evidence-based, and sustainable care for this population.

## Figures and Tables

**Table 1.** Summary of the impact of cognitive behavioral therapy on sleep quality and ISI (N=11)

First author (year)	Country	Sample size, age (year)	Intervention (regimen), follow-up, providers	Control (n)	Main outcome	Results	Author’s conclusion (quoted)
El-Monshed (2023) [18]	Egypt	88	CBT (group, 50–60 min, 7 sessions, n=43)	Routine care (n=45)	1) Sleep quality (PSQI)	1) MD, –2.73 [–3.50, –1.96], *p*<.001	“Group CBT for reducing levels of … insomnia while enhancing sleep quality in women going through menopause…”
		40–60	Follow-up: none		2) Sleep severity (ISI)	2) MD, –2.91 [–3.95, –1.87], *p*<.001	
			Researchers				
Yin (2023) [3]	China	60	CBT (offline, NR, 4 sessions, n=28)	Routine care (n=32)	1) Sleep quality (PSQI)	1-1) MD, –2.19 [–2.92, –1.46], *p*<.001	“Combined with cognitive-behavioral therapy is more effective in treating menopausal sleep disorders…”
		I: 46.6, C: 47.3	Follow-up: 24 wk			1-2) 24 wk: MD, –2.18 [–2.76, –1.60], *p*<.001	
			Experts		2) Sleep severity (ISI)	2-1) MD, –2.39 [–4.08, –0.70], *p*<.001	
						2-2) 24 wk: MD, –2.72 [–3.71, –1.73], *p*<.001	
Abdelaziz (2021) [15]	Egypt	80	CBT (Internet, 60 min, 6 sessions, n=40)	Routine care (n=40)	1) Sleep quality (PSQI)	1) MD, –2.63 [–3.69, –1.57], *p*<.001	“Internet‐based CBT programs... improving sleep quality and insomnia scores ...”
		53.1	Follow-up: none		2) Sleep severity (ISI)	2) MD, –2.90 [–4.40, –1.40], *p*<.001	
			Researchers				
Moradi Farsani (2021) [19]	Iran	44	CBT (group, 60 min, 6 sessions, n=22)	Routine care (n=22)	1) Sleep quality (PSQI)	1-1) MD, –3.83 [–5.12, –2.54], *p*<.001	“ CBT-I ... reducing their symptoms of insomnia and improving their quality of sleep”
		I: 51.4; C: 52.4	Follow-up: 10 wk			1-2) 10 wk: MD, –7.72 [–8.80, –6.64], *p*<.001	
			Expert		2) Sleep severity (ISI)	2-1) MD, –5.09 [–7.72, –2.46], *p*<.001	
						2-2) 10 wk: MD, –10.33 [–12.85, –7.81], *p*<.001†	
Ham (2020) [20]	Korea	44	CBT (individual, 30-60 min, 4 sessions booster counseling at 3 months, n=24)	Routine care (n=20)	1) Sleep quality (PSQI)	1-1) MD, –3.33 [–4.96, –1.70], *p*<.001	“Individual counseling combined with sleep hygiene education was effective in improving insomnia and sleep quality…”
		54.6	Follow-up: 52 wk			1-2) 52 wk: MD, –2.81 [–4.51, –1.11], *p*<.001	
			Expert		2) Sleep severity (ISI)	2-1) MD, –4.89 [–7.89, –1.89], *p*<.001	
						2-2) 52 wk: MD, –4.61 [–7.80, –1.42], *p*<.001	
Yang (2020) [21]	China	122	CBT (offline, NR, 8 sessions, n=61)	Routine care (n=61)	1) Sleep quality (PSQI)	1) NR	“CBT group had significantly higher sleep quality ...”
		I: 54.6; C: 54.4	Follow-up: none				
			Experts				
Drake (2019) [14]	USA	84	CBT (offline, NR, 6 sessions, n=41)	Routine care (n=43)	1) Sleep quality (CSD)	1-1) MD, –0.51 [–0.76, –0.26], *p*<0.001	“ CBT-I may be a superior treatment option for...insomnia”
		56.4	Follow-up: 24 wk			1-2) 24 wk: MD, –0.54 [–0.77, –0.31], *p*<.001	
			Expert		2) Sleep severity (ISI)	2-1) MD, –7.00 [–8.70, –5.30], *p*<0.001	
						2-2) 24 wk: MD, –6.49 [–8.62, –4.36], *p*<.001	
Green (2019) [22]	USA	49	CBT (group, 120 min, 12 sessions, n=28)	Routine care (n=21)	1) Sleep quality (PSQI)	1) MD, –3.79 [–6.58, –1.00], *p*<.001	“CBT-Meno was particularly effective in menopausal symptoms.”
		53.1	Follow-up: none				
			Expert				
Kalmbach (2019) [23]	USA	83	CBT (offline, NR, 6 sessions, n=42)	Routine care (n=42)	1) Sleep severity (ISI)	1) NR	“Postmenopausal insomnia patients with objective sleep disturbance appeared to have blunted treatment response to CBT…”
		56.4	Follow-up: 24 wk				
			Registered nurse				
Atema (2019) [24]	USA	254	1) CBT (offline, 60 min, 6 sessions, n=85).	Routine care (n=84)	1) Sleep quality (GSQS)	A1-1) MD, –2.25 [–3.70, –0.80], *p*<.001	“Internet‐based CBT programs had impact on sleep quality...”
		47.4	Follow-up: 24 wk			A1-2) 24 wk: MD, –2.10 [–3.55, –0.65], *p*<.001†	
			Experts			A2-1) MD, –1.51 [–2.96, –0.06], *p*<.001	
			2) (Internet, 60 min, 6 sessions, n=85)			A2-2) 24 wk: MD, –1.42 [–2.88, –0.04], *p*<.001†	
			Follow-up: 24 wk				
			Experts				
McCurry (2017) [25]	USA	88	CBT (telephone, 20–30 min, 6 sessions, n=47).	Routine care (n=41)	1) Sleep quality (PSQI)	1-1) MD, –3.10 [–4.24, –1.96], *p*<.001	“Telephone-based CBT-I ...improved sleep-... both immediately posttreatment and at 24 weeks of follow-up”
		I: 55.0; C: 54.7	Follow-up: 24 wk			1-2) 24 wk: MD, –0.54 [–0.77, –0.31], *p*<.001	
			Study coaches		2) Sleep severity (ISI)	2-1) MD, –6.40 [–7.07, –5.73], *p*<.001	
						2-2) 24 wk: MD, –5.20 [–6.27, –4.13], *p*<.001	

CBT: Cognitive behavior therapy; C: control group; CSD: consensus sleep diary; GSQS: Groningen Sleep Quality Scale; I: intervention group; ISI: Insomnia Severity Index; MD: mean difference; NR: not recorded; PSQI: Pittsburgh Sleep Quality Index

**Table 2.** Grouped summary of CBT-I intervention characteristics (N=11)

Characteristic	Category	n (%)	References
Mode of delivery	Face-to-face	8 (72.7)	[3], [14], [18], [19], [20], [21], [22], [23]
	Remote	2 (18.2)	[15], [24]
	Both	1 (9.1)	[25]
Number of sessions	4	2 (18.2)	[3], [20]
	6	6 (54.5)	[14], [15], [19], [23], [24], [25]
	7	1 (9.1)	[18]
	8	1 (9.1)	[21]
	12	1 (9.1)	[22]
Minutes/session	20–30	1 (9.1)	[25]
	30–60	1 (9.1)	[20]
	50–60	4 (36.4)	[15], [18], [19], [24]
	120	1 (9.1)	[22]
	Not reported	4 (36.4)	[3], [14], [21], [23]
Follow-up period	Immediate	4 (36.4)	[15], [18], [21], [22]
	6–10 weeks	1 (9.1)	[19]
	24 weeks	5 (45.5)	[3], [14], [23], [24], [25]
	52 weeks	1 (9.1)	[20]

CBT-I: Cognitive behavioral therapy for insomnia.

## Supplementary materials

Further details on supplementary materials are presented online (available at https://doi.org/10.4069/whn.2025.09.07).

Supplementary Table 1. Search strategies
