# Supplementary material for: Effects of cognitive behavioral therapy on sleep quality and insomnia severity index in women with menopausal insomnia: a systematic review and meta-analysis
Source: Womens Health Nurs. 2025 Dec 31;31(4):304–19. doi: 10.4069/whn.2025.09.07 (PMC12835450; doi:10.4069/whn.2025.09.07)
Supplement: Supplementary Table 1. — Search strategies [file whn-2025-09-07-Supplementary-Table-1.pdf]

Supplementary Table 1. Search strategies

Search database	Search terms	Search period	Search results
PubMed	#1 ("insomnia s"[All Fields] OR "sleep initiation and maintenance disorders"[MeSH Terms] OR ("sleep"[All Fields] AND "initiation"[All Fields] AND "maintenance"[All Fields] AND "disorders"[All Fields]) OR "sleep initiation and maintenance disorders"[All Fields] OR "insomnia"[All Fields] OR "insomnias"[All Fields]) AND (("sleep"[MeSH Terms] OR "sleep"[All Fields] OR "sleeping"[All Fields] OR "sleeps"[All Fields] OR "sleep s"[All Fields]) AND ("disease"[MeSH Terms] OR "disease"[All Fields] OR "disorder"[All Fields] OR "disorders"[All Fields] OR "disorder s"[All Fields] OR "disordes"[All Fields] OR ("problem"[All Fields] OR "problem s"[All Fields] OR "problems"[All Fields])))	24.10.28	178,505
	#2 "menopaus*"[All Fields] OR ("middle aged"[MeSH Terms] OR ("middle"[All Fields] AND "aged"[All Fields]) OR "middle aged"[All Fields] OR ("middle"[All Fields] AND "age"[All Fields]) OR "middle age"[All Fields])		5,013,763
	#3 "cognitive behavioral therapy"[MeSH Terms] OR ("cognitive behaviour therapy"[All Fields] OR "cognitive behavioral therapy"[MeSH Terms] OR ("cognitive"[All Fields] AND "behavioral"[All Fields] AND "therapy"[All Fields]) OR "cognitive behavioral therapy"[All Fields] OR ("cognitive"[All Fields] AND "behavior"[All Fields] AND "therapy"[All Fields]) OR "cognitive behavior therapy"[All Fields]) OR "CBT"[All Fields]		78,971
	#4 "sleep quality"[MeSH Terms] OR ("sleep"[All Fields] AND "quality"[All Fields]) OR "sleep quality"[All Fields] OR "PSQI"[All Fields] OR ("Pittsburgh"[All Fields] AND ("sleep quality"[MeSH Terms] OR ("sleep"[All Fields] AND "quality"[All Fields]) OR "sleep quality"[All Fields]) AND ("abstracting and indexing"[MeSH Terms] OR ("abstracting"[All Fields] AND "indexing"[All Fields]) OR "abstracting and indexing"[All Fields] OR "index"[All Fields] OR "indexed"[All Fields] OR "indexes"[All Fields] OR "indexing"[All Fields] OR "indexation"[All Fields] OR "indexations"[All Fields] OR "indexe"[All Fields] OR "indexer"[All Fields] OR "indexers"[All Fields] OR "indexs"[All Fields])) OR "VSH"[All Fields] OR ("Verran"[All Fields] AND "Snyder-Halpern"[All Fields]) OR ("isi"[Journal] OR "isi"[All Fields]) OR (("insomnia s"[All Fields] OR "sleep initiation and maintenance disorders"[MeSH Terms] OR ("sleep"[All Fields] AND "initiation"[All Fields] AND "maintenance"[All Fields] AND "disorders"[All Fields]) OR "sleep initiation and maintenance disorders"[All Fields] OR "insomnia"[All Fields] OR "insomnias"[All Fields]) AND ("sever"[All Fields] OR "severe"[All Fields] OR "severed"[All Fields] OR "severely"[All Fields] OR "severer"[All Fields] OR "severes"[All Fields] OR "severing"[All Fields] OR "severities"[All Fields] OR "severity"[All Fields] OR "severs"[All Fields]) AND ("abstracting and indexing"[MeSH Terms] OR ("abstracting"[All Fields] AND "indexing"[All Fields]) OR "abstracting and indexing"[All Fields] OR "index"[All Fields] OR "indexed"[All Fields] OR "indexes"[All Fields] OR "indexing"[All Fields] OR "indexation"[All Fields] OR "indexations"[All Fields] OR "indexe"[All Fields] OR "indexer"[All Fields] OR "indexers"[All Fields] OR "indexs"[All Fields]))		72,753
	#5 #1 AND #2 AND #3 AND #4		740
Cochrane	((insomnia) OR ((Sleep) AND ((Disorder) OR (Problem))) AND ((Menopaus*) OR (Middle age)) AND ((Cognitive behavioral therapy) OR (Cognitive behavior therapy) OR (CBT)) AND ((Sleep quality) OR (PSQI) OR (Pittsburgh Sleep Quality Index) OR (VSH) OR (Verran and Snyder-Halpern) OR (ISI) OR (Insomnia Severity Index))) in All text	24.10.28	482
Embase	#1 ('insomnia'/exp OR 'insomnia' OR ('sleep' AND (disoder OR problem)))	24.10.28	253,834
	#2 (menopaus* OR (middle AND age))		956,694
	#3 ('cognitive behavioral therapy' OR (cognitive AND behavior AND therapy) OR CBT)		108,212
	#4 ('sleep quality' OR psqi OR 'pittsburgh sleep quality index' OR vsh OR (verran AND snyder-halpern) OR isi OR 'insomnia severity index')		83,284
	#5 #1 AND #2 AND #3 AND #4		252
CINAHL	((insomnia) OR (((Sleep) AND ((Disorder) OR (Problem))) AND ((Cognitive behavioral therapy) OR (Cognitive behavior therapy) OR (CBT)) AND ((Sleep quality) OR (PSQI) OR (Pittsburgh Sleep Quality Index) OR (VSH) OR (Verran and Snyder-Halpern) OR (ISI) OR (Insomnia Severity Index)) in Full text	24.10.28	190
RISS	불면증 인지행동 수면의 질 수면 장애	24.10.28	
NDSL	"전체 = CBT-i AND 전체 = menopause AND 전체 = sleep quality AND 전체 = ISI OR PSQI"	24.10.28	
CNKI	((失眠症 OR 不寐病 OR 不寐 OR 失眠 OR 睡眠障碍) AND (绝经期 OR 绝经 OR 更年期)) AND (认知行为疗法 OR 认知行为)	24.10.31	8
VIP	((失眠症 OR 不寐病 OR 不寐 OR 失眠 OR 睡眠障碍) AND (绝经期 OR 绝经 OR 更年期)) AND (认知行为疗法 OR 认知行为)	24.10.31	1
Wanfagn	((失眠症 OR 不寐病 OR 不寐 OR 失眠 OR 睡眠障碍) AND (绝经期 OR 绝经 OR 更年期)) AND (认知行为疗法 OR 认知行为)	24.10.31	29
